# Immune Inflammation Pathways as Therapeutic Targets to Reduce Lethal Prostate Cancer in African American Men

**DOI:** 10.3390/cancers13122874

**Published:** 2021-06-09

**Authors:** Maeve Kiely, Stefan Ambs

**Affiliations:** Laboratory of Human Carcinogenesis, Center for Cancer Research, National Cancer Institute (NCI), National Institutes of Health (NIH), Bethesda, MD 20892, USA; maeve.bailey-whyte@nih.gov

**Keywords:** prostate cancer, African American, inflammation, health disparity

## Abstract

**Simple Summary:**

Men of African descent are twice as likely to die of prostate cancer than other men. While equal access to care is the key target to improve cancer survival, it is now known that there are differences in disease biology and risk factor exposure across population groups. These differences could be causatively linked to the existing prostate cancer health disparities. In this review, we will discuss the candidate role of inflammation and the immune response as contributing factors to the excessive burden of lethal prostate cancer among men of African ancestry. Furthermore, we will introduce the concept that these immunogenic vulnerabilities could be exploited to address the adverse outcomes experienced by these men. Lastly, we will summarize how these immunogenic and inflammatory differences could be targeted using current treatments to improve survival for men of African descent.

**Abstract:**

Despite substantial improvements in cancer survival, not all population groups have benefitted equally from this progress. For prostate cancer, men of African descent in the United States and England continue to have about double the rate of fatal disease compared to other men. Studies suggest that when there is equal access to care, survival disparities are greatly diminished. However, notable differences exist in prostate tumor biology across population groups. Ancestral factors and disparate exposures can lead to altered tumor biology, resulting in a distinct disease etiology by population group. While equal care remains the key target to improve survival, additional efforts should be made to gain comprehensive knowledge of the tumor biology in prostate cancer patients of African descent. Such an approach may identify novel intervention strategies in the era of precision medicine. A growing body of evidence shows that inflammation and the immune response may play a distinct role in prostate cancer disparities. Low-grade chronic inflammation and an inflammatory tumor microenvironment are more prevalent in African American patients and have been associated with adverse outcomes. Thus, differences in activation of immune–inflammatory pathways between African American and European American men with prostate cancer may exist. These differences may influence the response to immune therapy which is consistent with recent observations. This review will discuss mechanisms by which inflammation may contribute to the disparate outcomes experienced by African American men with prostate cancer and how these immunogenic and inflammatory vulnerabilities could be exploited to improve their survival.

Although cancer death rates have declined in the United States and other countries [1], disparities in cancer risk and outcomes persist, disproportionately affecting the systematically underserved and race/ethnic minoritized populations [2,3]. Prostate cancer is a key example of this with men of African descent in the United States and England continuing to have 2–3 times higher rates of fatal disease than other men [2]. Studies suggest that when there is equal access to care, survival disparities in prostate cancer are greatly diminished [4,5]. However, these investigations do not explain the notable differences in prostate cancer incidence, nor did they consider the now well-known differences in tumor biology across population groups. As shown recently, ancestral factors and disparate exposures may lead to distinct tumor biology in prostate cancer patients, resulting in a population-specific disease etiology [6,7,8,9]. While equal care remains the key target to improve survival, additional efforts should be made to gain comprehensive knowledge of the tumor biology in prostate cancer patients of African descent. Such an approach may identify novel intervention strategies for high risk groups in the era of precision medicine.

A growing body of evidence supports the hypothesis that inflammation plays a fundamental role in prostate cancer disparities. Key differences in activation of immune–inflammatory pathways between African American and European American men with prostate cancer are emerging and these biological processes may influence how African American men respond to therapy, as suggested by recent findings from clinical trials with the cancer vaccine, Sipuleucel T [10]. It is the aim of this review to discuss the candidate role of inflammation and the immune response as contributing factors to the excessive burden of lethal prostate cancer among men of African ancestry (Figure 1). Furthermore, we will introduce the concept that these immunogenic vulnerabilities could be exploited to address the adverse outcomes experienced by the high-risk African American population.

## 1. The Mutational and Immune–Oncologic Landscape of Prostate Tumors Differs between Populations

Prostate cancer displays large geographical differences in occurrence, with low incidence rates in East Asia and high rates in Western countries. Recognized risk factors for the disease include age, family history of the disease, race/ethnicity, and germline genetics [11,12,13,14]. It has been assumed that modifiable risk factors such as diet and lifestyle account for the majority of prostate cancers globally [15]. There is strong evidence from migration studies that the environment modulates prostate cancer risk [16,17]. Yet, there are few environmental factors that have consistently been linked to prostate cancer [11,18]. Notably, while prostate cancer is the leading cause of cancer death among men in many countries globally, sub-Saharan Africa and the Caribbean have more than double the age-standardized rates of mortality compared to other regions of the world, including North America and Europe [3,19]. This observation led to the hypothesis that ancestral factors may predispose men of sub-Saharan African ancestry to prostate cancer and a more aggressive disease [3,19]. Recent observations revealing the association of genetic ancestral factors with prostate cancer risk support this hypothesis [20,21,22,23,24]. Moreover, men of African ancestry are at an increased risk of developing fatal prostate cancer in the United States and England [2] and present with more aggressive disease in the Caribbean and sub-Saharan Africa [3,25]. The causes of the observed global prostate cancer health disparities are still being investigated but certainly include delayed diagnosis and lack of access to health care, ancestral, lifestyle, and environmental risk factors, and likely tumor biological differences [21,26,27].

Prostate cancer is a heterogeneous disease, in which inherited factors may account for about 40 to 50% of the cases [28]. Several familial susceptibility genes have been described, including *RNASEL*, *BRCA1*, *BRCA2*, and *HOXB13* [28,29,30,31,32]. *RNASEL*, or ribonuclease L, encodes a component of the interferon-regulated 2–5A system that functions in the antiviral roles of interferons [33], suggesting the importance of immune function in prostate cancer susceptibility. Most of the inherited risk for prostate cancer arises from common genetic variants [14]. More than 200 disease susceptibility loci are now known [24], but not all of them confer risk in men of African ancestry [34]. Numerous studies have examined the possibility of low penetrance genes contributing to the excessive burden of prostate cancer in African American men. To date, the best characterized risk locus for prostate cancer is located at 8q24. Multiple common variants within this locus increase the risk of prostate cancer in many populations [13,35,36,37,38]. As shown by several studies, this locus confers an even higher risk for prostate cancer in men of West African ancestry, when compared with men of European and East Asian ancestry, partly explained by variants that were only found in men of African ancestry [13,20,34,35,39,40]. Thus, the 8q24 region accounts for some of the excessive disease risk among men of African ancestry.

Prostate cancer can be classified into genomic subtypes, such as those with ETS-fusion gene arrangements and other subtypes that are negative for ETS-fusion gene arrangements, and either overexpress the *SPINK1* oncogene or carry *SPOP*, *FOXA1*, or *IDH1* mutations, or represent a triple-negative subtype (negative for *ERG*- and other *ETS*-fusions and *SPINK1*-negative) [6,41]. Early-stage prostate cancer contains few recurrent mutations in cancer-related genes (e.g., *ETS* gene fusions) [42,43]. Instead, prostate tumors are characterized by allelic gains of the *MYC* gene and deletions of the *NKX3–1*, *PTEN*, *Rb*, and *TP53* tumor suppressors [44]. Yet, there is strong evidence of prominent population differences in the acquisition of genetic alterations for prostate cancer. Reports showed that prostate tumors from patients of either European, African, or Asian descent exhibit notable differences in acquired chromosomal aberrations (e.g., *ERG* fusion and *PTEN* loss) and subtype distribution [6,7,8,9], indicating disparities in disease etiology and mutational events among these population groups. Comparing African American with European American patients [6,45,46,47], significant differences were observed in the frequency of *TMPRSS2–ERG* fusions (about 25% African American vs. 40–45% European American), *SPOP* mutations (about 20% African American vs. 10% European American), and *PTEN* deletions (about 10–15% African American vs. 30% European American). Chinese prostate cancer patients acquire mutations in *FOXA1* at a high frequency (about 40%), as shown by a recent report [9]. This gene is infrequently mutated in European-ancestry populations (<10%).

Chronic inflammation has been described as a prostate cancer risk factor that is associated with aggressive disease [48,49]. We found that aspirin use significantly reduces the risk of advanced prostate cancer in African American men [50]. Yet, no study has assessed whether these men commonly develop a systemic inflammatory process that increases the risk of prostate cancer progression and mortality. While environmental exposures, such as infections, promote systemic inflammation, ancestral factors may also influence inflammatory processes and the response to infections [51,52].

## 2. Inflammation as a Possible Driver of Aggressive Prostate Cancer in African American Men

The immune–inflammation signature that was initially described by Wallace et al. to be prevalent in prostate tumors of African American patients is central to the hypothesis that inflammation is a candidate driver of prostate cancer disparities [53]. Subsequently observed by others [54] and validated in TCGA [55], this signature includes upregulation of genes in the interferon (IFN) signaling pathway and contains elements of a viral mimicry signature. Further investigations of this signature in prostate tumors from African American men describe a signature which corresponds to a previously described “interferon-related DNA damage resistance signature”, also termed IRDS [56,57]. Detection of IRDS is a marker of decreased disease-free survival in prostate cancer and has been linked to acquired resistance to radiation and chemotherapy in breast cancer. Thus, upregulation of this signature in African American tumors indicates a mechanism by which either inflammatory ancestral factors or a yet unknown infectious agent may contribute adversely to prostate cancer outcomes. Even though the presence of IRDS in a tumor may indicate an adverse outcome, this signature may also constitute a vulnerability. Tumors with an interferon-stimulated gene signature were reported to be highly susceptible to inhibition of adenosine deaminase acting on RNA (ADAR1) [58,59].

Despite the fact that we know it occurs about twice as often in African American prostate tumors when compared to European American tumors [56], the precise origin of this immune inflammation signature remains unknown. However, presence of the signature is associated with an interferon-λ4 ΔG genotype [56]. This genotype is responsible for production of the interferon lambda 4 protein (IFNL4) and is most common in people of West African ancestry and influences host viral response [56,60]. In this context, the signature may have origins in either infection history [61], pro-inflammatory diets [62], changes to the epigenome [63], or reactivation of endogenous retroviral sequences which have been reported in African American prostate cancer patients [64].

Multiple studies reported upregulation of inflammatory mediators in the tumor microenvironment (TME) of African American prostate cancer patients, many of which have implications for disease prognosis [65,66,67,68,69]. Gillard et al. investigated the role of the stroma in prostate cancer disparities by isolating prostate fibroblasts from the TME of African American and European American men and culturing prostate cancer cell lines in conditioned fibroblast media [65]. They found enhanced expression of proinflammatory mediators including TrKB, BDNF, VEGF, and IL6 by tumor cells when the conditioned media was obtained from fibroblasts of African American origin as compared to European origin. This implicates the stromal environment in African American men as a potential driver of prostate cancer progression through elevation of inflammatory mediators. Weiner et al. report higher immune content in the TME of prostate tumors from African American men compared to European American men with the proportion of plasma cells contributing the greatest difference in quantity across three independent cohorts [68]. These high intra tumoral counts of plasma cells were further associated with increased metastasis-free survival in both a Johns Hopkins Medical Institute and the TCGA cohort, implicating plasma cells as candidate regulators of the immune responsiveness in African American men with prostate cancer. High plasma cell levels correlated with increased IgG expression and IFN signaling, and B cell and natural killer (NK) cell activity in tumors of these patients, showing a possible link between high plasma cells and increased immune activity. High IgG expression and NK cell activity also showed clinical significance as they were associated with increased metastasis-free survival. Our group previously detected a B cell signature in prostate tumors from current smokers, but smoking is thought to increase the risk of metastasis [70]. Collectively, these findings suggest a regulatory network between intratumor immune cells, inflammatory cytokines, and cells in the TME. Such a network, if clearly defined, may have potential as a biomarker of responsiveness to immunotherapy and targets to improve outcomes among African American patients.

In support of these findings, Awasthi and colleagues reported distinct changes in immune pathways including overall higher immune cell content, enrichment of immune oncological pathways, and lower DNA damage repair in prostate tumors of African American men compared to European American men [66]. After exploring discovery and validation cohorts of immune-related genes, the authors focused on 38 genes that were differentially expressed between the two population groups. Of these genes, 26 with the most robust gene expression differences were identified as being consistently associated with major immune biological pathways, including IFN signaling and cytokine signaling based on discovery and validation with two separate pathway analysis tools. As a stand-out, the proinflammatory gene *IFITM3* (IFN inducible transmembrane protein 3) was the only gene overexpressed in African American prostate tumors that predicted increased risk of biochemical recurrence only for African American men with prostate cancer, but not European American men.

The cause of this elevated immune–inflammation response is still under investigation. Numerous studies have shown that population differences in genetic ancestry can contribute to population differences in cancer susceptibility through processes that may involve inflammation. Genetic ancestry and natural selection are known to contribute to population differences in immune response to pathogens [52,71]. Furthermore, relationships of ancestry with expression levels of inflammatory cytokines are well documented in human populations [72,73]. As a modifiable risk factor, a pro-inflammatory diet that associates with high-grade prostate cancer is more commonly consumed by African American than European American men [62] and may lead to systemic inflammation. Other inducers of systemic inflammation may include stress exposures. Stress signaling transduces its biological effects through hypersecretion of the corticotrophin-releasing hormone and activation of the peripheral autonomic and sympathetic nervous system, which has direct effects on tumor biology and immune response, promoting inflammation, angiogenesis, mesenchymal differentiation, and metastasis [74]. As a final example, co-morbidities including chronic infections and diabetes can be excessively high in African American men [75,76,77]. They are frequently associated with increased inflammatory processes which could contribute to cancer development. This suggests that there could be a role for both biological and environmental factors in the elevated immune–inflammation pathways that are reported in the prostate tumors from men of African descent, as previously discussed [78].

## 3. African American Men May Have a Differential Response to Certain Therapies for Metastatic Prostate Cancer

**Figure 2:**
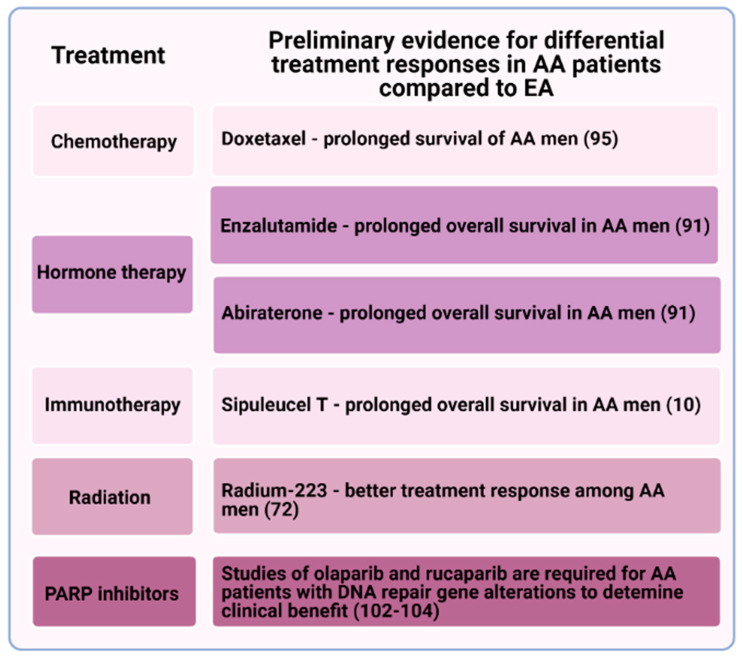
Preliminary evidence for certain treatment response differences between African American (AA) and European American (EA) patients with metastatic castration-resistant prostate cancer (mCRPC). Current approved treatment modalities for mCRPC, how clinical responses may differ between the two patient groups, and where additional studies are warranted. For PARP inhibitor use, the pathologic role of germline variants of unknown significance in DNA repair genes that commonly occur in AA men needs to be investigated.

The peer-reviewed literature now provides some evidence that men of African descent may respond differently across the gamut of both standardized and emerging options of care for prostate cancer, including radiation, hormone therapy, chemotherapy, and immunotherapy. Differences in immune response may play a key role in many of these observations. Metastatic castration-resistant prostate cancer (mCRPC) is a main cause of lethal prostate cancer and therefore remains a key focus for research. Despite patients with mCRPC having multiple treatment options targeting a variety of mechanisms (Figure 2), median overall survival is still only around 3 years [79]. This further highlights the need for inclusion of diverse biospecimens in scientific studies and historically understudied populations in clinical trials to determine who is benefitting optimally from these currently approved treatments.

## 4. Radiation

Radium-223 is an approved therapeutic option for mCRPC patients with symptomatic bone metastases. Zhao et al. examined the response to radium-223 treatment in men from a Veteran Affairs cohort with mCRPC [80]. With equal access to care across the cohort, this group found that African American men may have a better response to this treatment compared to European American men, resulting in a 25% decreased risk of mortality in this equal access to care study. African American men in this study were more likely to have received docetaxel beforehand and the improved response to therapy was despite the African American cases being more likely to not start radium treatment until further along in the disease course. Patients harboring DNA damage repair mutations have prolonged overall survival after radium-223 treatment compared to patients who do not have these alterations [81,82,83]. This is also the subject of another clinical trial currently in the recruitment stage (NCT04489719). With Awasthi et al. reporting decreased DNA damage repair capacity in prostate tumors of African American men, it can be speculated that inactivating mutations that decrease the DNA damage repair capacity in tumors from African American men may contribute to the positive outcomes post treatment with radium-223 [66].

A recent, small, phase II trial (NCT02463799) found combining radium-223 treatment with Sipuleucel-T increased progression-free survival and overall survival in men with mCRPC [84]. Now that studies have shown better responses from African American men treated with radium-223 and Sipuleucel-T separately [10,80], a planned larger trial may inform on whether African American men may benefit synergistically from this combination approach.

## 5. Immunotherapy

Immunotherapy has not been as successful in treating prostate cancer as with other hematologic or solid cancers and clinical trials show a modest [85] to no effect [79,86,87] on survival. This has been attributed to prostate cancer not being as immunogenic as other cancers. However, recent studies indicate a potential role for immunotherapy in certain patient groups with prostate cancer. Precision medicine strategies targeting immunotherapy to those men with the best response is the preferred goal. Evidence is currently being built to support the hypothesis that African American men may have a differential and perhaps superior response to certain treatments due to changes in immune cell response and a differing tumor biology.

Tumors from men of African descent may have a heightened response to immunotherapies, and specifically to cancer vaccines, as assumed from the presence of an interferon signature in their tumors and increased immune content in the TME [56,66]. Studies have shown that young people who self-report as African American mounted an increased immune response to vaccination [88,89]. Sartor et al. recently reported that African American men with mCRPC who were treated with the cancer vaccine, Sipuleucel-T, in the PROCEED trial/registry, had significantly better survival than the European American patients [10]. Median overall survival was 35.3 months for African American men compared to 25.8 months for European American men, in a PSA-matched set. This difference became even greater when measured in patients with a baseline PSA below the median, with median overall survival of 54.3 months in African American men versus 33.4 months in European American men. Increased activation of dendritic cells is a proposed mechanism of action of the vaccine and in agreement with this, activated dendritic cells in localized tumors have subsequently been associated with improved distant metastasis free survival [90]. Mechanistically, evidence points towards a complex interplay of immune cells with tumor biology which may predict prognosis and response to therapy. However, the lack of tumor specimens from African American men means that more work must be done to capitalize on the differences in the immune landscape which may improve response to treatment in this population.

Generally, poor immunogenicity has resulted in little success for PD-L1 blockade in treatment of prostate cancer [87,91]. This has been attributed in part to relatively low PD-L1 expression from tumor cells [92,93]. However, this is not consistent across the literature, with studies also reporting increased PD-L1 expression and association with biochemical recurrence [94] and shorter metastasis free survival [95]. Petitprez et al. provide preliminary evidence that a composite assessment of both PD-L1 and CD8 expression in localized prostate cancer may be a good strategy for predicting outcomes in mCRPC [95]. A group in Norway reported high PD-L1 expression in post-prostatectomy, hormone-naïve tumor epithelial cells with a non-significant trend towards an inverse association between PD-L1 expression and biochemical failure-free survival [96]. However, clinical trials investigating the effect of PD-L1 inhibition reported no significant clinical benefit. Yet, they have typically not included men of African descent [87].

Recent work has focused on PD-L1 expression on tumor-infiltrating immune cells. Bishop et al. reported enzalutamide-resistant prostate cancer patients showing increased PD-L1 expression on dendritic cells and high PD-L1 T cells when compared to enzalutamide-sensitive or treatment-naïve patients [97]. African American ethnicity and an aggressive cancer phenotype have been associated with prediction of tumor PD-L1 positivity in hormone-naïve tumors [98], suggesting a potential benefit for immunotherapy in African Americans at high risk of aggressive disease, but this has not been replicated yet [66]. When tumors are enzalutamide-sensitive, McNamara et al. preliminarily reported increased overall survival for African American, chemotherapy-naïve men with mCRPC treated with abiraterone or enzalutamide compared to European American men [99]. Overall survival was 918 days for African Americans compared to 781 days for European Americans. This study in a Veteran Affairs population was retrospective in design, again pointing to the value of equal access to care across populations. Thus, additional work is warranted, including measurement of PD-L1 in tumor samples from African American men post various treatment regimens to account for increased immunogenic response to therapy.

## 6. Other Treatment Opportunities

Historically, participation of African American men in clinical trials has been low. Reasons for this are multifactorial but include historical mistrust of the medical profession as a result of systemic racism and major ethical breaches in the past [100]. A higher prevalence of comorbidities and a lack of access to academic medical centers involved in trials may also prevent access to trials [100,101,102]. This prevents generalizability across population groups when reporting clinical trial data. A recent example highlights the need to include diverse population groups and possibly stratify clinical trial participants by race to get a fuller picture of treatment response. Halabi et al. completed a meta-analysis of survival outcomes for African American versus European American men in phase III clinical trials treating mCRPC with docetaxel [103]. With just 6% of African American participants, they reported that while overall median survival was similar, a pooled hazard ratio of 0.81 (95% CI, 0.72 to 0.91) post adjustment for baseline prognostic factors estimated that African American men may have a significantly decreased risk of death compared to European American men. This was despite African American men having baseline characteristics known to be prognostic of overall survival including statistically significantly worse performance status, higher testosterone levels, higher PSA levels, and lower hemoglobin levels.

It is assumed by many that the prostate cancer biology in men of African ancestry is intrinsically more aggressive—at least for a subset of patients—leading to a survival health disparity in the population [104]. Yet, this does not mean that African American men would not respond as well as European American men to most standard therapies. In fact, it appears that the treatment responses of African American and European American men are mostly similar. Yet, tumors in African American men could still respond better to certain therapies compared to the average response among European American men. Some of the treatments or combination treatments discussed in this review were the subject of small clinical trials and so these therapeutic options might not be widely offered yet in the clinic. Therefore, the findings require further evaluation in larger studies but do suggest that there is a potential role for these treatments in reducing the survival disparities observed in prostate cancer. African American men are less likely to be recruited into clinical trials and may not have the opportunities to avail of these new therapeutic options.

## 7. Germline and Somatic Mutations in DNA Repair Pathways

A proposed feature of prostate tumors in African American men that may play a prominent role in differential response to treatment is a deficiency in DNA damage repair capacity. Both germline and somatic alterations to DNA damage repair pathways have now been found in prostate tumors across multiple studies [66,105,106,107]. Tumors from African American men were reported to have a significantly lower level of DNA repair capacity when compared to those from European American men. Notably, these tumors seemed to have an increased radiosensitivity [66].

Germline mutations in DNA repair genes have a higher occurrence in metastatic prostate cancer when compared to localized prostate cancer [105,106]. BRCA1/2 pathogenic variants have been associated with more aggressive prostate cancer and adverse survival outcomes [108,109]. DNA repair gene mutations may contribute to aggressive disease in African American men. Acquired somatic mutations may differ among patient groups, with Yadav et al. reporting that prostate tumors from African American men were twice as likely to have at least one mutation in nucleotide excision repair pathway genes compared to European American (89% vs. > 40%) [107]. Petrovics and colleagues reported that germline variants in DNA repair genes of unknown significance had an increased frequency in African American men (4.6%) compared to European American men (1.6%) [110]. As the significance of these is undetermined, there is an opportunity to investigate whether they play a pathogenic role in prostate cancer. The same authors also reported that just 0.7% of men with localized prostate cancer carried pathogenic variants of BRCA1/2 mutations, but this increased over 4-fold to 3.1% in patients with metastatic and advanced disease, indicating that the presence of known BRCA1/2 pathogenic variants is linked to disease status [110]. Because the FDA-approved PARP inhibitors, olaparib and rucaparib, have shown success in prolonging overall survival in mCRPC patients with mutations in these DNA damage response genes [111,112,113], they should be made available to all African American men with prostate cancer who carry these mutations. 

Altered DNA damage repair pathways may sensitize tumors to immunotherapeutic approaches. Several clinical trials across many cancer sites including metastatic prostate cancer are currently underway, targeting DNA damage repair-deficient tumors with checkpoint inhibitors (extensively reviewed by Bever et al.) [114]. Mechanistically, in prostate cancer, the stimulator of the IFN genes (STING) pathway has been linked to the recruitment and activation of interferon-related genes in vitro, increasing sensitivity to the immune checkpoint inhibitor PD-L1 in DNA repair-deficient tumors [115,116,117,118]. As a low DNA repair capacity may increase tumor genomic instability and tumor mutational burden, this again might constitute a vulnerability to immunotherapeutic strategies [119]. It has been suggested that enhancement of this genomic instability through use of radiation, chemotherapy, or PARP inhibitors could augment the immunotherapeutic response [114,120,121].

## 8. Anti-Inflammatory Drug Aspirin for Prevention of Adverse Outcomes in African American Men with Prostate Cancer

While the research community plays catch-up with ensuring proper representation of population groups in clinical trials and precision medicine studies, strategies for the prevention of lethal prostate cancer and reduction in adverse outcomes are of paramount importance to men of African descent who continue to experience a disproportionally high mortality from prostate cancer. An extensive body of evidence including both preclinical and clinical studies led the United States Preventative Services Task Force to recommend the anti-inflammatory drug aspirin for prevention of colorectal cancer for an albeit narrow category of adults [122,123,124]. In keeping with the hypothesis that inflammation is one of the drivers of the prostate cancer disparities, our group explored the link between regular use of aspirin and prostate cancer in African American men. We found that regular aspirin use significantly reduces the risk of both advanced prostate cancer and disease recurrence in these men [50]. The finding is consistent with a similar observation in a previous study [125]. Inhibition of the pro-inflammatory cyclooxygenase/thromboxane A2 pathway has been identified as a potential mechanism of action for aspirin in the prevention of metastatic cancer [126]. Using a retrospective cohort, we found a distinct association between high urinary 11-dehydrothromboxane B2 (the stable metabolite of thromboxane A2) and aggressive prostate cancer as well as adverse survival outcomes for African American men. Importantly, our ongoing research showed high 11-dehydrothromboxane B2 was inversely correlated with aspirin use, indicating a potential benefit of aspirin in preventing lethal prostate cancer through inhibition of TXA2 synthesis.

Lastly, data prospectively obtained in the Southern Community Cohort Study suggested that aspirin use is tentatively associated with a reduced prostate cancer mortality in African American men [127]. Hurwitz et al. also observed this inverse relationship between aspirin use and prostate cancer mortality in both African American and European American men using the ARIC cohort [128], again pointing to the potential benefit of aspirin use for men at high risk of fatal prostate cancer.

## 9. Conclusions

Elevated inflammatory processes in African American men with prostate cancer are a candidate biological driver of disparate disease risks. There is a need for more clinical trials specifically focused on the treatment response of African American men with metastatic prostate cancer [129]. Improved inclusion of minority populations in trials is essential to further enhance our knowledge of how inflammation and the immune response and alterations to molecular pathways may govern the response to emerging therapies across all patient groups. With evidence now building that suggests increased clinical benefit with certain therapies among African American men when compared to European American men, targeting inflammatory processes and the immune system could be an important strategy to reduce lethal disease in high-risk populations such as men of African ancestry.

## Figures and Tables

**Figure 1:**
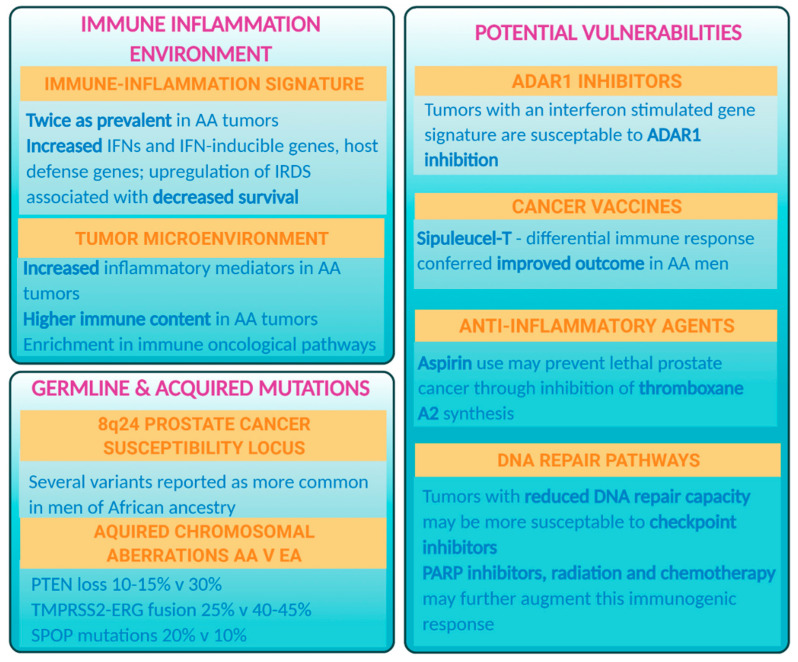
Key differences in the tumor immune–inflammation environment and the mutational spectrum between African American and European American men with prostate cancer. These differences open up potential vulnerabilities which preliminary studies have indicated could be exploited with treatment options, with some of them having demonstrated favorable responses in African American men.

## Data Availability

The study is a literature review and did not analyze any data.
